# The influence of marital status and race/ethnicity on risk of mortality for triple negative breast cancer

**DOI:** 10.1371/journal.pone.0196134

**Published:** 2018-04-26

**Authors:** Carol Parise, Vincent Caggiano

**Affiliations:** Sutter Institute for Medical Research, Sacramento, California; Sudbury Regional Hospital, CANADA

## Abstract

**Purpose:**

To assess the effect of marital status and the role of race/ethnicity on breast cancer specific mortality in women with triple negative breast cancer (TNBC).

**Methods:**

The study utilized the California Cancer Registry to identify 22,812 cases of first primary female TNBC. Unadjusted Kaplan-Meier breast cancer specific survival was computed. Cox Proportional Hazards modeling was used to compute the adjusted risk of breast cancer specific mortality for women who were single, separated, divorced, and widowed when compared with women who were married. Models were adjusted for age, stage, tumor grade, SES, and treatment with surgery, chemotherapy, hormone therapy, and radiation therapy. Hazard ratios (HR) and 95% confidence intervals (CI) were reported.

**Results:**

Separated (HR: 1.45; 95% CI: 1.14–2.01) and widowed (HR: 1.39; 95%CI: 1.23–1.57) white women had a higher risk of mortality than white married women whereas single and divorced white women had the same risk of mortality. For Asian/Pacific Islanders (API), only single (HR: 1.55; 95% CI: 1.17–2.06) and divorced (HR:1.81; 95% CI:1.26–2.60) women had a higher risk of mortality than married women. Marital status had no influence on risk of mortality for either black or Hispanic women.

**Conclusions:**

The risk of mortality associated with marital status is dependent on race/ethnicity. Only white and API women with TNBC have a marital advantage.

## Introduction

Breast cancer is the most common cancer among women but it is a heterogeneous disease and there is variability in both incidence and survival among breast cancer subtypes. Triple negative breast cancer (TNBC), characterized as being negative for estrogen receptor (ER), progesterone receptor (PR), and human epidermal receptor growth factor 2 (HER2), is the second most common breast cancer subtype and has the worst prognosis. [1–3]

When all breast cancer subtypes are combined and breast cancer is considered a single disease, tumor characteristics such as stage at diagnosis and tumor grade as well as social factors including race/ethnicity and marital status have been found to be associated with both breast cancer incidence and survival. Disparities in the incidence and mortality of breast cancer among white, African American, Hispanic, Asian, and American Indian women have been well-documented. [4–8] Studies of individual breast cancer subtypes have shown that TNBC is more common in young women, black and Hispanic women, and women of lower socioeconomic status (SES) when compared with the ER+/PR+/HER2- subtype, the most common breast cancer subtype. [9, 10]

Marriage has been found to have a survival advantage for many cancers including breast cancer. [11–15] However, it is unknown whether this advantage applies to all breast cancer subtypes. The purpose of this study is to assess the effect of marital status on breast cancer specific mortality in women with TNBC and evaluate the role of race/ethnicity on this association.

## Methods

The study utilized the California Cancer Registry (CCR) to identify 22,812 cases of first primary female TNBC diagnosed between January 1, 2000 and December 31, 2014 and reported to the CCR as of December 31, 2015. (ICDO-3 sites C50.0-C50.9) [16] Cases had complete data for tumor size, grade, American Joint Commission on Cancer (AJCC) stage of diagnosis, surgery (lumpectomy, mastectomy), chemotherapy, hormone therapy, radiation therapy, cause of death, age, socioeconomic status (SES), marital status, and race/ethnicity. This research study involved analysis of existing data from the CCR without subject identifiers or intervention. Therefore, the study was categorized as exempt from institutional review board oversight.

The determination of mortality, ER, PR, HER2, race/ethnicity, and socioeconomic status are the same as reported in many of our previous publications [7, 9, 17–23] Cases were reported to the Cancer Surveillance Section of the California Department of Public Health from hospitals and other facilities providing care or therapy to cancer patients residing in California. [24] Breast cancer-specific mortality was defined as a death due to breast cancer as documented by the codes ranging from C50.01 to C50.91 of the International Statistical Classification of Diseases and Related Health Problems, 10^th^ Revision. [25]

ER and PR status were recorded according to pathologists' interpretation of the assays. ER and PR were considered negative if immunoperoxidase staining of tumor cell nuclei was less than 5%. ER and PR status may also have been determined by examining cytosol protein. ER was considered negative if there were fewer than 3 femtomoles per milligram of cytosol protein and PR was considered negative if there were fewer than 5 femtomoles per milligram of cytosol protein. [16]

HER2 was assessed through immunohistochemistry (IHC) or fluorescence in situ hybridization (FISH). IHC is scored on a qualitative scale from 0 to 3+, based on interpretation of staining intensity, with 0 through 1+ classified as negative, 2+ as borderline, and 3+ as positive. [26] FISH was scored on a quantitative scale with less than 2 copies of the HER2 gene classified as negative and two or more copies as positive. [27]

Race was based on information obtained from the medical record which was derived from patient self-identification, assumptions based on personal appearance, or inferences based on the race of the parents, birthplace, surname, or maiden name. For the present study, race/ethnicity was classified into four mutually exclusive categories: non-Hispanic white, African American or black, Hispanic, and Asian/Pacific Islander.

SES was derived using data from the 2000 US census for cases diagnosed from 2000 through 2005, and the American Community Survey was used for cases diagnosed from 2006 through 2014. [28]This SES variable is an index that utilizes education, employment characteristics, median household income, proportion of the population living 200% below the Federal Poverty Level, median rent and median housing value of census tract of residence for case and denominator population. A principal component analysis was used to identify quintiles of SES ranging from 1 (the lowest) to 5 (the highest).[29] This area based SES measure has been used in many studies utilizing cancer registry data.[9, 17, 30–36]

Marital status was defined at the time of diagnosis as married, single/never married, separated, divorced, and widowed.

### Statistical analysis

Contingency tables were used to evaluate the distribution of age, stage, subtype, tumor size, tumor grade, race/ethnicity, treatment, SES, and race/ethnicity for each classification of marital status. Difference in mean age by marital status was compared using analysis of variance and post hoc tests.

Kaplan–Meier survival analysis and the Log-Rank test were used to compare unadjusted survival rates by marital status for each race/ethnicity. Cox Proportional Hazards modeling was used to compute the risk of mortality for women who were single, separated, divorced, and widowed when compared with women who were married. The race by marital status interaction was tested to determine if the risk of mortality associated with marital status depended on race. Models were adjusted for age, stage, tumor grade, SES, and treatment with surgery (lumpectomy, mastectomy), chemotherapy, hormone therapy, and radiation therapy. Variables were considered statistically significant and HRs were interpreted only when the Wald *Χ*^2^ was p< 0.05.

All analyses were performed using IBM SPSS 21.0. [37]

## Results and discussion

Over 50% of white, Hispanic, and API women were married and 39% of black women were married. Thirty-two percent of black women were single compared with less than 20% for all other ethnicities. There was an inverse association in stage at diagnosis for single and married women. The percent of single women increased with increasing stage at diagnosis and the opposite was true for married women. (Table 1)

**Table 1:** Demographic and clinicopathologic characteristics of 22,812 cases of triple negative first primary female breast cancer from the California Cancer Registry 2000–2014.

	Single/Never Married N = 3,834	Married N = 13,365	Separated N = 353	Divorced N = 2,591	Widowed N = 2,669	Total N = 22,812
Mean age (years) ±SD	51.38±13.25	55.02±12.71	51.83±11.19	58.32±11.91	74.03±11.25	56.96±14.11
Age						
<46	1,325 (25.9%)	3,284 (64.2%)	103 (2.0%)	378 (7.4%)	24 (0.5%)	5,114
46–69	2,149 (16.3%)	8,229 (62.5%)	223 (1.7%)	1,755 (13.3%)	817 (6.2%)	13,173
70+	360 (8.0%)	1,852 (40.9%)	27 (0.6%)	458 (10.1%)	1,828 (40.4%)	4,525
Race/ethnicity						
White	1,745 (13.6%)	7,773 (60.1%)	130 (1.0%)	1,564 (12.2%)	1,691 (13.1%)	12,863
Black	860 (32.1%)	1,037 (38.7%)	68 (2.5%)	412 (15.4%)	303 (11.3%)	2,680
Hispanic	935 (19.0%)	2,945 (59.9%)	130 (2.6%)	480 9.8%)	453 (8.6%)	4,913
Asian/Pacific Islander	294 (12.5%)	1,650 (70.0%)	25 (1.1%)	135 (5.7%)	252 (10.7%)	2,356
AJCC Stage						
1	1,055 (13.4%)	4,870 (62.0%)	89 (1.1%)	876 (11.2%)	962 (12.3%)	7,852
2	1,916 (17.7%)	6,338 (58.4%)	185 (1.7%)	1,233 (11.4%)	1,182 (10.9%)	10,854
3	661 (20.4%)	1,764 (54.6%)	62 (1.9%)	372 (11.5%)	374 (11.6%)	3,233
4	202 (23.1%)	363 (45.0%)	17 (1.9%)	110 (12.6%)	151 (17.3%)	873
Tumor Grade						
Well differentiated; Grade 1	77 (12.7%)	352 (58.0%)	4 (0.7%)	68 (11.2%)	106 (17.5%)	607
Moderately differentiated;Grade 2	540 (13.8%)	2,226 (57.0%)	37 (1.7%)	438 (11.4%)	663 (10.4%)	3,904
Poorly differentiated; Grade 3	3,087 (18.7%)	10,382 (58.2%)	294 (2.6%)	2,009 (10.9%)	1,833 (9.6%)	17,605
Undifferentiated; Grade 4	130 (16.8%)	405 (58.6%)	18 (1.5%)	76 (11.4%)	67 (11.7%)	696
Socioeconomic Status(SES)						
SES 1-Lowest	827 (24.7%)	1,583 (47.2%)	89 (2.7%)	428 (12.8%)	427 (12.7%)	3,354
SES 2	805 (19.2%)	2,253 (53.7%)	85 (2.0%)	547 (13.0%)	504 (12.0%)	4,194
SES 3	788 (16.5%)	2,760 (57.8%)	65 (1.4%)	607 (12.7%)	553 (11.6%)	4,773
SES 4	758 (14.5%)	3,187 (61.1%)	67 (1.3%)	575 (11.0%)	631 (12.1%)	5,218
SES 5-Highest	656 (12.4%)	3,582 (67.9%)	47 (0.9%)	434 (8.2%)	554 (10.5%)	5,273
Chemotherapy	2,887 (18.0%)	10,008 (62.2%)	281 (1.7%)	1,819 (11.3%)	1,085 (6.7%)	16,080
Radiation therapy	1,713 (15.8%)	6,625 (61.3%)	170 (1.6%)	1,243 (11.5%)	1,057 (9.8%)	10,808
Endocrine therapy	119 (15.5%)	433 (56.5%)	15 (2.0%)	89 (11.6%)	110 (14.4%)	766
Surgery						
None	279 (26.8%)	493 (47.4%)	24 (2.3%)	135 (13.0%)	110 (10.6%)	1,041
Lumpectomy	1,932 (15.9%)	7,283 (60.0%)	182 (1.5%)	1,425 (11.7%)	1,309 (10.8%)	12,131
Mastectomy	1,623 (16.8%)	5,589 (58.0%)	147 (1.5%)	1,031 (10.7%)	1,250 (13.0%)	9,640

**Fig 1:**
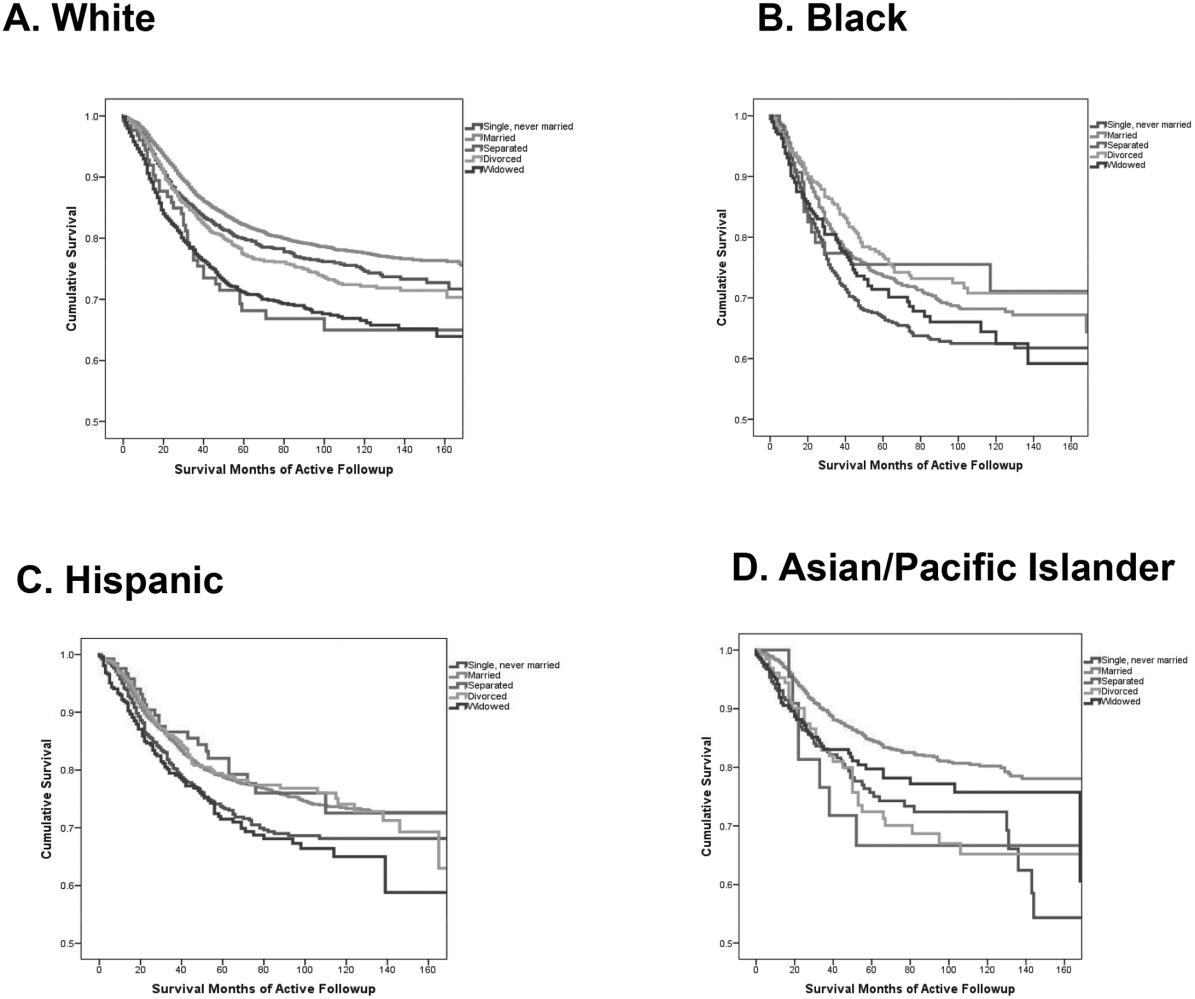
Unadjusted Kaplan-Meier breast cancer specific survival for single, married, separated, divorced, and widowed women with TNBC who were white (Panel A), black (Panel B), Hispanic (Panel C), and Asian/Pacific Islander (Panel D).

Results of the Cox Regression analysis indicated that the race *X* marital status interaction was statistically significant (*χ*^2^ df = 12 = 31.05, p = 0.002). Therefore separate models were computed for each race/ethnicity so that Kaplan-Meier survival statistics, hazard ratios (HR), and 95% confidence intervals (CI) assessed differences between women of the same race. Fig 1. shows the Kaplan-Meier breast cancer specific survival graphs according to marital status for white (Panel A), black (Panel B), Hispanic (Panel C), and API (Panel D) women.

A marital advantage was apparent but differences were noted within the race/ethnicities. Married white women had superior survival over single (p = 0.006), separated (p < 0.001), divorced (p < 0.001), and widowed (p < 0.001) white women. Married black women only had better survival than single black women (p = 0.003). Married Hispanic women had better survival than both single (p = 0.002) and widowed Hispanic women (p < 0.001). Married API women had better survival than all except for separated API women (p = 0.067).

Table 2 shows the HRs and 95% confidence intervals for women who were single, separated, divorced, or widowed when compared with married women of the same race. Separated and widowed white women had a higher risk of mortality than white married women whereas single and divorced white women had the same risk of mortality. For APIs, only single and divorced women had a higher risk of mortality than married women. Marital status had no influence on risk of mortality for either black (*X*^2^_4_ = 2.06; p = 0.726) or Hispanic (X^2^_4_ = 5.94, p = 0.204) women.

**Table 2:** Hazard ratios (95%CI) for 22,812 white, black, Hispanic, and Asian/Pacific Islander women with triple negative breast cancer. Hazard ratios are adjusted for age, stage, grade, socioeconomic status, and treatment.

	White n = 12,863	Black n = 2,680	Hispanic n = 4,913	Asian/Pacific Islander n = 2,356
	HR (95%CI)	HR (95%CI)	HR (95%CI)	HR (95%CI)
**Married**	1.00	1.00	1.00	1.00
**Single/Never Married**	1.12 (0.99–1.27)	*	*	1.55 (1.17–2.06)
**Separated**	1.45 (1.14–2.01)	*	*	1.97(0.92–4.23)
**Divorced**	1.11 (0.97–1.25)	*	*	1.81 (1.26–2.60)
**Widowed**	1.39 (1.23–1.57)	*	*	1.31 (0.90–1.90)

*Unadjusted and adjusted Wald *Χ*^2^ was not statistically significant for Black and Hispanic women (p > 0.05)

Marriage has been found to be advantageous for cancer survival for both men and women. [38–41] Studies of the association of marital status with breast cancer survival have shown that younger, unmarried women are diagnosed with breast cancer at later stages and are more likely to die of the disease than married women. [12, 15, 42–47]

The so called marital advantage has been found to vary by race/ethnicity. [48] Martinez [46] and colleagues found that all-cause mortality in cancer patients was higher in unmarried versus married patients but risk of mortality was worse for unmarried white women than for unmarried API women. Simon and Severson [49] noted that marriage did not improve the relative risk of dying of breast cancer in African American versus white women and Weider et al. [50] reported that African American race was a poor prognostic indicator for breast cancer survival independent of marital status.

Although socioeconomic status (SES) is a known risk factor for survival for several types of cancer [9, 20, 33] it appears that marital status is an independent risk factor. [51] The most likely link between marriage and breast cancer survival is social support. Several studies suggest that a strong social network is associated with better survival. [52–58] Family, friends, and spouses motivate each other to seek medical care which can lead to an earlier cancer diagnosis. [12, 59] They also encourage their family and friends with cancer to follow up with their treatments and seek support from other breast cancer survivors. [60]

Conversely, stress and social isolation have been found associated with poorer health for breast cancer survivors. [61–65] Cancer patients with lower levels or loss of perceived support are at a higher risk for mortality. [39, 66] These studies suggest that if presence of a supportive spouse predicts lower mortality, [56] then women with breast cancer in stressful marriages would not benefit from the marital advantage.

Most studies of breast cancer survival combine all subtypes. However, incidence, survival, and prognostic factors vary among the breast cancer subtypes defined by ER, PR, and HER2. [18] We chose to exclusively study TNBC because it is the most lethal subtype and there are racial/ethnic differences in survival. Black and Hispanic women are more likely to be diagnosed with and die from TNBC than white women. [9, 67, 68]

Our results concur with studies that found that married women with breast cancer fare better than unmarried women. However, when adjusting for sociodemographic and tumor characteristics, marriage is only an advantage for white and API women, the races least likely to have TNBC, whereas black and Hispanic women with TNBC have no advantage if they are married. [1–3, 9, 10]

The present study was designed to compare the risk of mortality associated with marital status of women with TNBC within a single race/ethnicity so that the hazard ratios represented single, separated, divorced, and widowed women compared with married women of the same race/ethnicity. This makes it difficult to compare our findings to those of the literature because to our knowledge, this is the first study that assessed breast cancer mortality for a single subtype within four race/ethnicities.

Why do black and Hispanic women with TNBC not have a marital advantage? There is some evidence that black women have lower marital satisfaction than white women which may partially explain our results. [69–71] We found no evidence in the literature to support that Hispanic women have less satisfactory marriages than white women but our findings concur with Delgado et al [72] who found no difference in risk of breast cancer mortality in married versus unmarried Hispanic women. Future investigations that measure perceived marital support by race/ethnicity would be necessary to provide insight on the results of the present study.

Retrospective, population-based studies using cancer registry data have limitations. The determination of ER, PR, and HER2 were performed by a wide variety of laboratories without testing inter-rater reliability. Treatment information from the CCR lacks specific information regarding drug type and dose. The classification of race/ethnicity could have influenced the results, especially for the Asian/Pacific Islander group. Previous publications found marked variability within Asian/Pacific Islanders in both incidence and survival of the ER/PR/HER2 subtypes when this group was split into seven Asian categories versus combing them all into one. [7, 21]. It is possible that the results among Asian/Pacific Islander women may have been different had we stratified this category into seven groups. However, we did not have sufficient cases to break the category down any further since we were only including cases of TNBC.

Despite these limitations, a study of over 22,000 cases of triple negative breast cancer allows for stratified analysis of race/ethnicity and provides real world insight.

## Conclusion

The risk of mortality associated with marital status is dependent on race/ethnicity. White and API women but not black and Hispanic women with TNBC have a marital advantage.
